# A functional signal profiling test for identifying a subset of HER2-negative breast cancers with abnormally amplified HER2 signaling activity

**DOI:** 10.18632/oncotarget.12480

**Published:** 2016-10-05

**Authors:** Yao Huang, David J Burns, Benjamin E Rich, Ian A MacNeil, Abhijit Dandapat, Sajjad M. Soltani, Samantha Myhre, Brian F Sullivan, Leo T Furcht, Carol A Lange, Sara A Hurvitz, Lance G Laing

**Affiliations:** ^1^ Celcuity LLC, Minneapolis, MN 55446, USA; ^2^ Department of Laboratory Medicine and Pathology, University of Minnesota, Minneapolis, MN 55455, USA; ^3^ Division of Hematology, Oncology and Transplantation, Departments of Medicine and Pharmacology and The Masonic Cancer Center, Minneapolis, MN 55455, USA; ^4^ Division of Hematology/Oncology and Jonsson Comprehensive Cancer Center, University of California, Los Angeles, CA 90095, USA

**Keywords:** HER2 negative, HER2 signaling, targeted therapy, CELx HSP test, tumor primary cells

## Abstract

The results of clinical trials evaluating the efficacy of HER2 inhibitors in patients with breast cancer indicate that the correlation between HER2 receptor levels and patient outcomes is as low as 50%. The relatively weak correlation between HER2 status and response to HER2-targeting drugs suggests that measurement of HER2 signaling activity, rather than absolute HER2 levels, may more accurately diagnose HER2-driven breast cancer. A new diagnostic test, the CELx HER2 Signaling Profile (CELx HSP) test, is demonstrated to measure real-time HER2 signaling function in live primary cells. In the present study, epithelial cells extracted fresh from breast cancer patient tumors classified as HER2 negative (HER2^−^, *n* = 34 of which 33 were estrogen receptor positive) and healthy subjects (*n* = 16) were evaluated along with reference breast cancer cell lines (*n* = 19). Live cell response to specific HER2 agonists (NRG1b and EGF) and antagonist (pertuzumab) was measured. Of the HER2^−^ breast tumor cell samples tested, 7 of 34 patients (20.5%; 95% CI = 10%–37%) had HER2 signaling activity that was characterized as abnormally high. Amongst the tumor samples there was no correlation between HER2 protein status (by cell cytometry) and HER2 signaling activity (hyperactive or normal) (Regression analysis *P* = 0.144, R^2^ = 0.068). One conclusion is that measurement of HER2 signaling activity can identify a subset of breast cancers with normal HER2 receptor levels with abnormally high levels of HER2 signaling. This result constitutes a new subtype of breast cancer that should be considered for treatment with HER2 pathway inhibitors.

## INTRODUCTION

Breast cancer classification is largely based upon a patient's expression levels of estrogen receptor (ER) and human epidermal growth factor receptor 2 (HER2).

HER2 is normally expressed on all breast epithelial cells, but HER2 gene (*ERBB2*) amplification and/or HER2 protein overexpression is detected in approximately 15%–20% of breast cancers and associated with more aggressive disease progression, metastasis, and a poorer prognosis [1–4]. HER2 clinical status is most commonly determined using immunohistochemistry (IHC) to assess HER2 protein expression or fluorescent *in situ* hybridization (FISH) to assess *ERBB2* amplification.

Agents targeting HER2, such as trastuzumab, lapatinib, and pertuzumab, significantly improve clinical outcomes in HER2^+^ patients [4, 5]. Currently, a patient's eligibility for HER2-targeted therapies is determined by their IHC- or FISH-based HER2 testing scores [4]. However, results from recent studies and trials have cast doubt on the conventional opinion that only patients with HER2^+^ tumors benefit from HER2 targeted therapies. Paik and colleagues reported that in the NSABP B-31 trial, 174 of 1787 patients (9.7%) originally classified as HER2^+^ were actually HER2^−^ when their HER2 status was reanalyzed in a central laboratory. Surprisingly, these HER2^−^ patients benefited as much from adjuvant trastuzumab as the HER2^+^ patients. The authors concluded that there was no significant correlation between *ERBB2* copy number and trastuzumab benefit [6]. These intriguing results have led to the ongoing NSABP B-47 trial, which is testing the addition of trastuzumab to standard chemotherapy in HER2^−^ tumors.

The molecular mechanisms underlying the clinical benefit of HER2 targeted therapy in the adjuvant setting in HER2^−^ patients whose tumors do not display classical HER2 overexpression or amplification remain unclear. One emerging hypothesis is that aberrant HER2 signaling, rather than increased HER2 expression, may account for the clinical benefit of HER2 targeted therapy in some HER2^−^ breast cancers. It has been well established that in breast tumors, the catalytically inactive HER family member HER3 is an obligate partner for HER2 and couples active HER2 to the PI3K/AKT pathway to drive tumor cell growth and survival [7–9]. Recent data also suggests that expression of NRG1b, a cognate ligand for HER3 and HER4, is associated with HER2 activation in HER2^−^ tumors [10]. These findings suggest that increased HER2 expression may not be an absolute requirement for increased HER2 signaling activity.

Current HER2 tests using either IHC or FISH do not provide a functional status of HER2 or its signaling network, which may significantly lessen these tests' effectiveness in patient selection for HER2 signaling inhibitors, particularly for HER2^−^ patients. Thus, tests to identify HER2^−^ patients who may benefit from HER2 targeted therapies represents an unmet medical need.

Biosensor-based methods have provided data for real-time live cell assessment of signaling activities and responses to drugs in cancer cell lines [11–13]. The aim of the present study was to provide early evidence that a biosensor test had the necessary sensitivity and reproducibility for clinical utility and that this test could provide proof-of-concept that some breast cancers classified as HER2^−^ by IHC may exhibit abnormally elevated HER2 activity. The present study uses an impedance biosensor in a 96-well microplate format monitoring cell adhesion properties to quantify HER2 signaling activity in the CELx HER2 Signaling Profile test (CELx HSP) [11]. To measure the signaling driven specifically by HER2 participation, EGF and NRG1b initiated signaling is measured with and without the presence of pertuzumab. The monoclonal antibody, pertuzumab, has been demonstrated to bind to HER2 and prevent HER2 dimerization essential for its participation in ligand driven signaling. The result reported from the CELx HSP test thus represents the amount of ligand driven HER2 signaling activity directly associated with HER2 heterodimerization with HER3 and HER1.

The findings presented here suggest that a subset of HER2^−^ breast cancer patients whose cells display abnormal HER2 signaling activity as measured by the CELx HSP test may be candidates for HER2 signaling inhibitors.

## RESULTS

### Growth of epithelial cells derived from patient tissue specimens

**Figure 1:**
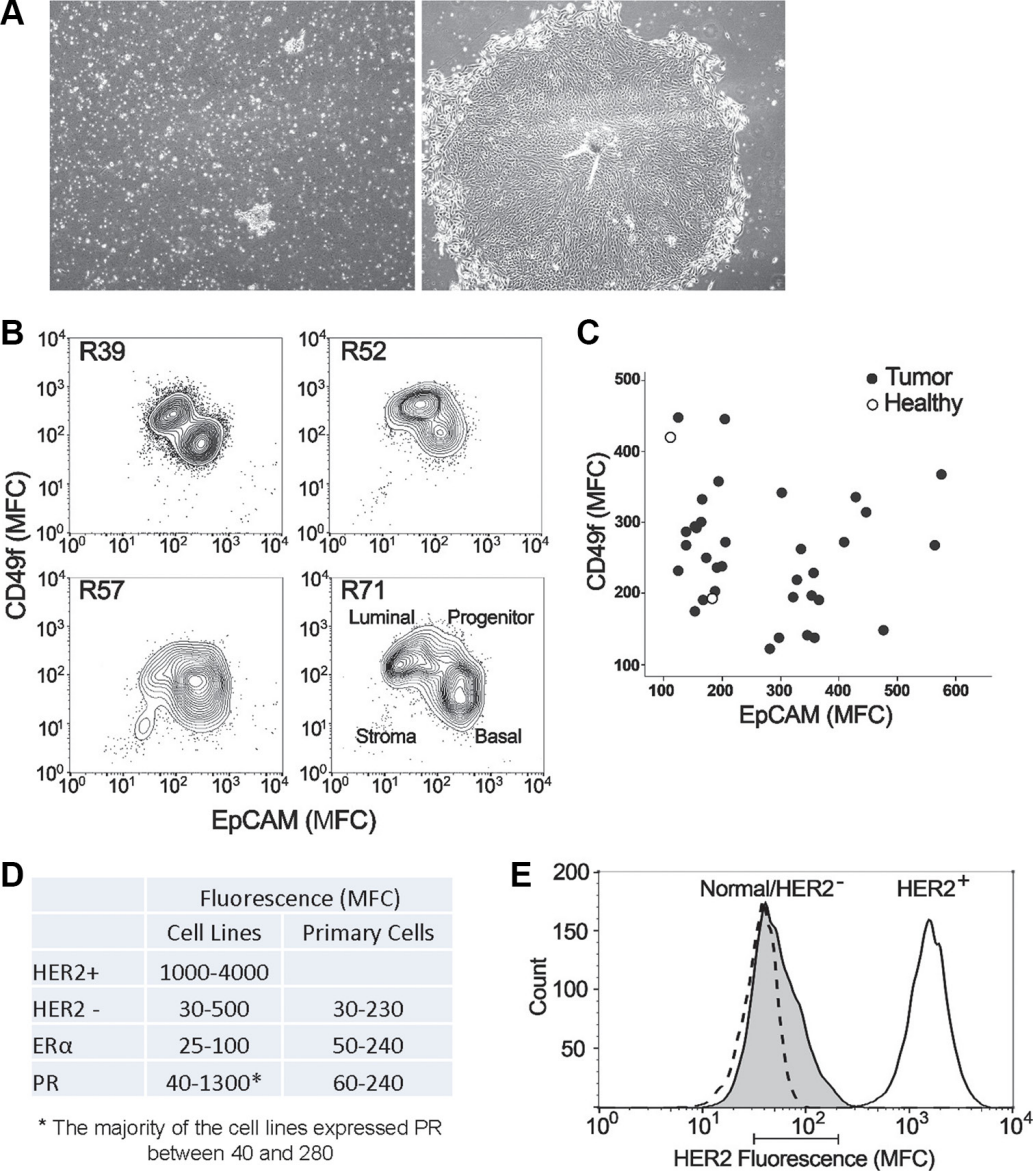
Characteristics of primary epithelial cells derived from patient tissue specimens (**A**) A representative culture of primary cells from a digested tumor biopsy at day 1 (a) and day 5 (b) of culture, respectively. (**B**) Flow cytometric analysis of luminal (EpCAM^+^, Claudin4^+^) and basal (CD49f^+^, CD10^+^) markers on four representative tumor primary cells harvested at the time of CELx HSP test. (**C**) Plot showing the Mean Fluorescence Channel (MFC) of the luminal marker EpCAM (x-axis) and the basal/progenitor biomarker CD49f (y-axis) for all 34 tumor samples tested (*filled circles*). For comparison, 2 healthy samples are displayed (*empty circles*). (**D**) Comparison of expression levels of HER2, ERα, and PR between primary cells and cell lines (HER2^+^ and HER2^−^), which were measured by flow cytometry. (**E**) Histogram plot of HER2 expression measured by flow cytometry of a representative tumor (shaded peak) compared to HER2+ cell line SKBR3 (solid line) and HER2- cell line MDA231 (dashed line) that is coincidentally in the same range as the healthy samples. The bar below the graph represents the range of means from all the primary tumors and healthy tissue tested (MFC range 31-210).

Tissue specimens were digested and placed into serum-free mammary epithelial media. Cells and cell clusters attached to the 6-well plate surface within 24 hours and colonies became visible within four to six days of culture. The majority of the colonies appeared phenotypically epithelial, marked by a tight cobblestone appearance (Figure 1A). Fibroblasts were occasionally present in very low numbers but their presence became less evident after a few days as the epithelial cell number expanded. Figure 1B shows flow cytometry results for four typical primary samples with > 95% heterogeneous epithelial (luminal, myo-, and stem/progenitor) and less than 1% fibroblasts. Figure 1A shows representative cultures from a digested tumor specimen at day 1 and day 5 of culture, respectively. Cells were monitored for growth and harvested when they approached ~50% confluence (~2 × 10^5^ to 3 × 10^5^ cells). A fraction of harvested cells was used for flow cytometric analysis. Of the 34 tumor specimens analyzed here, 33 were reported as Luminal A or B [14, 15] and one was triple negative (ER^−^/PR^−^/HER2^−^) (Table 1).

**Table 1:** HER1-3 expression in tissue specimen-derived primary tumor cells determined by flow cytometry

Sample #	Genotype	HER1	HER2	HER3
R131	ER+, HER2–, PR+	104	105	140
R160	ER+, HER2–, PR–	65.8	96.2	151
R20	ER+, HER2–, PR+	93.3	99.8	173
R22	ER+, HER2–, PR+	229	101	158
R23	ER+, HER2–, PR+	216	97	188
R25	ER–, HER2–, PR–	171	105	14
R35*	HER2–	12.4	28.9	51.3
R36*	HER2–	130	117	131
R37*	HER2–	146	87.4	164
R39*	HER2–	N/A	201	N/A
R40*	HER2–	34.2	42.7	88.1
R41	ER+, HER2–, PR+	54.8	113	624
R42	ER+, HER2–, PR+	214	106	150
R43	ER+, HER2–, PR+	141	30.6	171
R45	ER+, HER2–, PR+	82.8	151	503
R47*	HER2–	38.1	163	266
R49*	HER2–	107	73.7	115
R51	ER+, HER2–, PR+	373	116	83.7
R52	ER+, HER2–, PR+	28.4	36.2	84.7
R53	ER+, HER2–, PR+	41.6	163	327
R54	ER+, HER2–, PR+	159	56.5	107
R56	ER+, HER2–, PR+	185	115	209
R57	ER+, HER2–, PR+	111	178	361
R58	ER+, HER2–, PR+	45.4	96.4	311
R60	ER+, HER2–, PR+	32.2	47.7	88.7
R66	ER+, HER2–, PR+	156	72.5	104
R69	ER+, HER2–, PR+	248	144	195
R71	ER+, HER2–, PR+	60	33.1	179
R79	ER+, HER2–, PR+	186	114	129
R82	ER+, HER2–, PR+	95.2	129	148
R84	ER+, HER2–, PR+	165	74.2	165
R91	ER+, HER2–, PR-	318	99.3	117
R95	ER+, HER2–, PR+	85.2	166	465
R99	ER+, HER2–, PR+	119	46.1	63
R62	Healthy example	111	100	141
SKBr3	HER2+ Cell Line (DAKO 3+)	47.8	2386	290
	**HER2^−^ Tumor Range**	360.6	172.1	610
	**HER2 - Mean**	125.9	163.6	190.2
	**HER2^−^ Tumor Max**	373	201	624
	**HER2^−^ Tumor Min**	12.4	28.9	14

* Indicates ER/PR data not available.

ER+/HER2- are classical luminal A and luminal B intrinsic subtypes.

Highlighted samples are test positive, high signaling.

Next, the harvested cells were characterized using a set of biomarkers that define mammary epithelial subsets [16]. When used in combination, these markers identified four cell populations found in the normal human mammary gland, including EPCAM^+^CD49f^−^ epithelial cells, EPCAM^+^CD49f^+^ luminal progenitor cells, EPCAM^−^CD49f^+^ basal/myoepithelial cells, and EPCAM^−^CD49f^−^ stromal cells. Figure 1B shows a flow cytometric analysis of four representative tumors at the time of CELx HSP test, indicating populations containing both luminal-like and basal-like characteristics. Figure 1C shows a plot of all the tumors (*n* = 34) with respect to CD49f (basal marker) and EPCAM (luminal marker) indicating that the method used to grow the epithelial cells allowed for the growth of a diverse set of tumor cells. As the majority of tumor samples were from ER^+^/PR^+^ patients, ER and PR were expected to be expressed in the primary cells grown from these tumors, as shown in Figure 1D.

### Patient tissue-derived primary cells did not overexpress HER2

Protein expression of HER receptors in samples derived from cancer (*n* = 34) and healthy (*n* = 16) subjects were assessed by flow cytometry; all primary mammary epithelial cells evaluated in this study expressed normal amounts of HER2 (Figure 1D). Figure 1E is a histogram plot of HER2 expression measured by flow cytometry of a representative tumor compared to the HER2^+^ breast cancer cell line SKBR3 (DAKO test score 3+) and the HER2^−^ breast cancer cell line MDA-MB-231 (DAKO test score 0). The bar below the histogram plot represents the range of means from all the primary tumors and healthy tissue tested. As in Figure 1D and 1E, all 34 tumor and 16 healthy samples had normal/low HER2 expression levels in the range of HER2^−^ cell lines. This confirms the clinical test results reported to us for each specimen by a clinical pathologist using standard IHC clinical protocols, that the 34 tumor samples were true HER2^−^. HER1 and HER3 were also present at various levels on all the cells tested, demonstrating that these proteins were expressed in each cell sample and available for hetero-dimerization with HER2 (Table 1).

### The CELx HSP test for patient-derived primary cells

The CELx HSP test measures HER2-related signaling in live breast cancer cells in real-time by evaluating the difference between agonist (ligand/growth factor)-induced signals in the absence or presence of a HER2 dimerization antagonist over a 4-hour duration [11]. The current study first aimed to demonstrate that the CELx HSP test could meet the analytical signal sensitivity and pathway specificity expectations for a clinical diagnostic using patient-derived primary cells.

**Figure 2:**
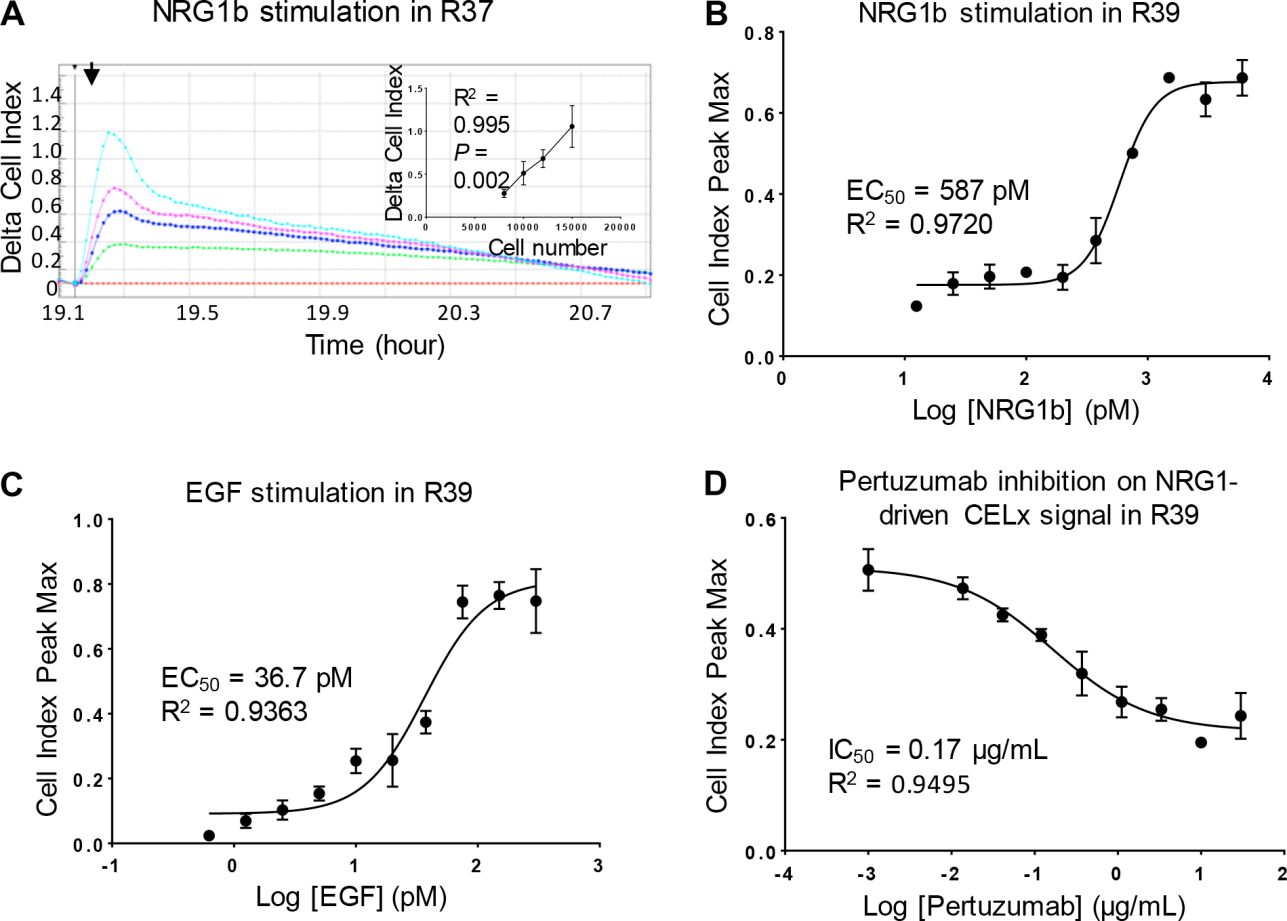
Optimization and specificity of CELx HSP test in primary cells (**A**) R37 primary cells seeded at different densities (8000, 10000, 12000, 15000 cells per well) in a sensor plate were stimulated with NRG1b (3 nM). CELx curves are displayed using Delta CI values to demonstrate the relative signals normalized to the time point (*arrow*) when NRG1b was added. Positive correlation between cell number and NRG1-driven CELx signal is shown in the inset. (**B** and **C**) Dose-response curves of NRG1b and EGF stimulation of CELx signals in R39 primary cells. (**D**) Dose-response curve of pertuzumab showing its specific inhibitory effect on NRG1b-driven CELx signal.

The seeding density for primary cells on the sensor plates was first studied to optimize the cell number that would result in a robust ligand-induced CELx HSP signal. Results indicated that 1.5 × 10^4^ cells per well gave a consistent ligand-induced signal across many different cell samples and showed the largest proportional increase in the CELx HSP time-course signal. Representative results of NRG1b-induced CELx HSP time-course signal using primary cells from one study subject (R37) are shown in Figure 2A.

HER2 hetero-dimerizes with HER1, HER3, and HER4, to activate ligand dependent signaling in conjunction with these HER family members. Thus the ligands NRG1b (binds HER3 and HER4), EGF (binds HER1), and a HER2 receptor dimerization blocker (pertuzumab) were employed to demonstrate that the CELx HSP signals are attributable to HER2 signaling through HER1 and HER3. The EC_50_ dose for each stimulus, NRG1b and EGF, was first determined. It was demonstrated that the magnitude of the CELx HSP signals correlated with the dose of each ligand (NRG1b and EGF) and that the curve fit values obtained were in close agreement with literature [17]. Examples of dose-response curves of NRG1b and EGF in a representative primary cell sample (R39) are shown in Figure 2B and 2C, respectively.

Tests were then performed to assess whether the ligand-driven CELx HSP HER2 signals could be inhibited by pertuzumab, an anti-HER2 monoclonal antibody that specifically blocks HER2 dimerization with HER1, HER3, and HER4 and thus allows the assessment of the proportions of HER2-dependent signals driven by HER2- hetero-dimerizations and HER family homo-dimerizations in the quantitation of the CELx HS*P value*s [17, 18]. As shown in a representative sample in Figure 2D, pertuzumab inhibited NRG1b-initiated CELx HSP signal with an IC_50_ of 0.17 ug/mL. Taken together, these results demonstrate that the CELx HSP test can specifically detect ligand-induced HER2-related signals and determine whether a HER2-driven test signal is sensitive or insensitive to a HER2-targeted drug. A conservative 10 μg/mL (~68 nM) pertuzumab concentration that was consistent wth drug dose exposure in patients was selected. This concentration provided ~100% inhibition on the NRG1b-induced CELx HSP signal in test development samples and reduced the chance for off-target effects. The pertuzumab concentration and inhibition result were in agreement with the results of others [17] and 5-fold below the mean trough serum concentration reported in pertuzumab's FDA BLA (www.accessdata.fda.gov/drugsatfda_docs/nda/2012/125409Orig1s000MedR.pdf).

### CELx HSP test using HER2^−^ primary tumor cells

After the test demonstrated sensitivity and specificity, the test was used with HER2^−^ patient tumor cells to determine whether a sub-group of samples had abnormal HER2 pathway signaling. CELx HSP tests were performed on 34 tumor cell samples from patients with breast cancer classified as HER2^−^ to measure HER2 pathway stimulation and signal specificity. For comparative purposes, 16 primary breast epithelial cell samples from healthy patients, and DAKO IHC test standard breast cancer cell lines SKBr3 and MDA-231 were also subjected to CELx HSP Tests.

**Figure 3:**
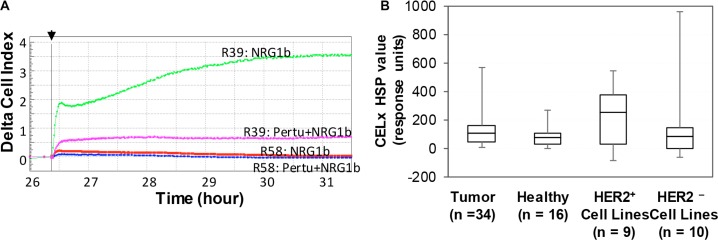
Identification of subgroups of HER2^−^ tumor-derived primary cells by CELx HSP test (**A**) Representative CELx time-course curves representing a high, abnormal HER2 signaling activity in a high responder (R39) and a low HER2 signaling activity in a non-responder (R58). In this display, curves of NRG1 stimulation in the absence versus presence of pertuzumab (10 μg/mL) are presented. (**B**) Box-and-whisker plots of the CELx HSP test scores for four cell sample groups (HER2^−^ patient-derived tumor cells and cells derived from healthy tissue are plotted with HER2^+^ cell lines, HER2^−^ cell lines [11].

NRG1b-induced and EGF-induced CELx HSP signals for tumor and healthy primary cells and a HER2^+^ reference cell line (SKBr3) are summarized in Table 2. CELx HSP curves representing primary tumor samples that have high (R39) and low (R58) HER2 signaling activities are shown in Figure 3A. Note that the high NRG1b responder has more than 10 times greater signal than the low responder, indicative the test has a large dynamic range.

**Table 2:** CELx HSP test results of 8 HER2- patient samples with healthy and HER2+ cell line

HER2-Negative Patient Samples	Total NRG1 Signaling	Total EGF Signaling	Total HER- dependent Signaling	Total HER2-dependent Signaling from NRG1	Total HER2- dependent Signaling from EGF	Test Measurand (*Total HER2- dependent Signaling*)	CELx Test Result
R39	634	294	928	475	88	563	Abnormal
R20	539	286	824	409	120	529	Abnormal
R160	349	229	578	332	99	430	Abnormal
R82	336	332	668	272	40	312	Abnormal
R95	250	116	366	227	44	271	Abnormal
R25	326	206	533	238	29	267	Abnormal
R71	336	211	547	228	23	252	Abnormal
R22	8	353	361	1	78	79	Normal
R62 Healthy	32	13	46	31	7	38	Normal
SKBR3 cell line	802	367	1169	401	143	544	Abnormal

Box-and-whisker plots of the CELx HSP test scores were constructed for the four groups (HER2^+^ cell lines, HER2^−^ cell lines, HER2^−^ patient-derived tumor cells, and cells derived from healthy tissue) presented in Figure 3B. The plot demonstrates the relative quartile distributions of the four groups by CELx HSP test score. Note for the box-and-whisker plot that the median HER2 activation levels are comparable for the tumors clinically classified as HER2^−^, HER2^−^ cell lines, and the healthy patient samples. Whereas, the median of the ligand-induced HER2 activity in HER2^+^ cell lines is approximately 2.5-fold greater than the median of the other groups.

A cutoff of 250 response units (90% of the upper range of the healthy responses and coincidental with the median value of the HER2^+^ cell line population) was determined to represent an abnormally active HER2 signaling network in primary breast cancer cells. Previous work using this cutoff criteria demonstrated that only 4 out of 9 (44%) of the HER2^+^ cell lines are considered highly active for HER2 signaling [11]. Only one of the healthy patient samples had a CELx HSP test response level slightly greater than 250.

Of the HER2^−^ breast tumor cell samples tested, 7 of 34 patients (20.5%; 95% CI 10.0–37.1) had HER2 signaling activity that was characterized as abnormally high. Given that none of these primary tumor and healthy cells have elevated HER2 expression levels (Figure 1D and 1E), these results indicate that the robust HER2-driven signalling observed in the primary tumors is not due to the HER2 overexpression.

### CELx HSP test positive patients are not identifiable by patient and tumor clinical characterizations and HER family fluorescence cytometry

All seven (7/34) of the breast cancer specimens that were CELx HSP test positive for upregulated HER2-related signaling were from patients with invasive/infiltrative carcinoma and 1/7 patients were diagnosed with lobular carcinoma. CELx HSP test positive patients in this group were all grade 2/grade 3 and only one of the patients was node positive. The small number of patients did not allow for statistical analysis of correlation of clinical characterizations with CELx HSP test results. The fluorescence cytometry data provided no key to determining which samples would have CELx HSP Test positive results. The fluorescence cytometry results show that HER1 expression in all seven CELx HSP test positive samples was below the mean and median for this group of 34 tumor samples. HER2 protein level in the tumor sample group showed no correlation with CELx HSP test positive group result even within the HER2^−^ group (*P* = 0.144, R^2^ = 0.068). In the seven CELx HSP test positive HER2^−^ samples, the HER3 expression levels span from low to high range (Table 1). Retrospective analysis of HER2^−^ patient sample data collected in parallel to the CELx HSP test results provided no insight as to which patients would have hyperactive HER2 signaling.

## DISCUSSION

When patients are inherently resistant to a targeted therapy, the specific disease mechanism the targeted therapy is designed to inhibit may not be present despite the presence of the target, the drug, and a correlative genomic biomarker. This highlights the importance of developing tools that provide more precise diagnosis of a patient's disease mechanism than is possible with current biomarker-based tests.

Accurate determination of HER2 status is critical for optimizing employment of HER2 targeted therapies and improving clinical outcomes in patients with HER2-driven breast cancer. However, IHC and FISH tests do not provide a quantitative measurement of HER2 function (e.g., the status of the HER2 signaling network), which is perhaps the most crucial aspect underlying the pathophysiology of HER2-driven diseases.

In the present study, the potential use of the CELx HSP test was investigated to quantitatively assess the dynamic status of the HER2 signaling network in patient-derived live primary breast tumor cells from patients classified as HER2^−^ by traditional IHC and or FISH-based clinical pathology tests.

The present work first established that cultured primary cells derived from fresh patient tumor tissue were of the right type and would consistently maintain a level of phenotypic diversity reminiscent of the tumor tissue. The cells derived from these tissues continue to express ER (26/34 samples; one sample was ER^−^/PR^−^/HER2^−^) as measured by intracellular flow cytometry. Several biomarkers that define luminal and basal types of epithelial cells were also used [16]. The tumors maintained multiple phenotypically distinct subsets (see Figure 2A) of epithelial cells suggesting a phenotypically diverse set of tumors.

Next, the work demonstrated that HER2+ patients were not inadvertently included in the study and that there were no HER2+ cells present in the test samples. Only samples reported as HER2 IHC 0 or 1+ in the original patient pathology report were used in this study. The HER2 status was independently confirmed by FACS and RT-qPCR and found to be 100% concordant with the pathology reports. Figure 1E demonstrates good dynamic range and sensitivity of FACS to the presence of any HER2+ cells. The concordance level found between the IHC testing performed at the treating institution and FACS and RT-q-PCR testing for IHC 0 and 1+ tumors was consistent with a number of studies evaluating the concordance between IHC results and FISH amplification status (~99%). However, concordance between IHC and FISH results are lower for IHC 2+ results (~90%–95%) and even lower for IHC 3+ results (~85%) suggesting the potential need for improved methods of evaluating HER2 receptor status [19–21]. New HER2 mRNA quantification methods are emerging that may offer improved specificity or sensitivity than IHC or FISH methods [22].

HER2+ samples were specifically excluded from this study. The current test was developed to detect the ligand dependent HER2-driven cancer the authors believe is most likely to be found in a normal HER2 expression level breast cancer patient. Future work may demonstrate the CELx HSP test can possibly be applied to HER2+ breast cancer patients to identify patients not likely to respond to current anti-HER2 therapies such as trastuzumab. Since the effectiveness of trastuzumab is reported to involve Antibody Dependent Cell-mediated Cytotoxicity (ADCC) killing in HER2+ cells, and the present CELx HSP Test does not effectively assess ADCC killing, additional development would be required.

The tissue samples used to establish the primary cultures were taken directly from a specimen evaluated first by a pathologist at the treating institution. The pathology reports for 11 of 34 samples used in this study noted some presence of Ductal Carcinoma *In Situ* (DCIS) in addition to breast cancer tissue. There is no well-established way to distinguish DCIS cells from tumor cells outside of a pathological stained tissue cross-section slide. For this reason, the authors were not able to independently confirm that only tumor cells were included in the cell samples tested here. In the 11 specimens obtained from patients reported to have DCIS in addition to breast cancer, nearly every section described in the pathology reports was described as having > 90% ER+/PR+ nuclear positivity, suggesting the vast majority of the specimen evaluated was cancer tissue. Two of the 11 samples obtained from patients with DCIS were HSP+, the same proportion of HSP+ samples not reporting DCIS +. While not definitive evidence of the lack of DCIS cells or the lack of potential bias from the presence of DCIS cells in our test samples, the equal proportion of HSP+ patients in the patients with DCIS and without DCIS is suggestive that no bias exists in our sample set due to potential presence of DCIS cells. In the event that DCIS cells were included in the test sample, it has been suggested that abnormal HER2 in DCIS is indicative of patients more likely to progress to invasive carcinoma [23].

After the sample type was confirmed, a complete CELx HSP test was performed on 34 breast cancer samples originally classified by a clinical pathologist as HER2^−^ and 16 healthy control samples. The test identified a subset (7 of 34, 20.5%) of HER2^−^ primary tumor cell samples that had abnormally elevated HER2 signals above the median level of HER2^+^ cell lines. The CELx HSP test positive patients could not be identified by fluorescence flow cytometry analysis of any HER family markers. Several previous studies describe the potential to use HER3 expression levels to predict breast cancer prognosis [24–27]. Importantly, Bae et al. in a study of 950 cases with long term follow-up data did not find HER3 expression levels correlated to prognosis in hormone receptor positive breast cancer. The present study, which studied predominantly hormone receptor positive breast cancer tumors (26 of 35 tumors), supports this conclusion. Statistical analysis of our CELx HSP test results demonstrates that there is no correlation between HER2 receptor expression level and HER2 signaling. Taken together, these findings provide strong evidence that hyper-activation of HER2 signaling occurs primarily through NRG1b stimulation in HER2^−^ samples and that these patients may benefit from HER2 signaling inhibitors.

The results of the recent NSABP B-31 trial comparing the safety and efficacy of doxorubicin and cyclophosphamide followed by paclitaxel (AC-T) to that of AC-T plus trastuzumab suggested that some patients with HER2^−^ cancer benefited as much from HER2-targeted adjuvant trastuzumab as patients with HER2^+^ [6]. It has been speculated that patients with HER2^−^ cancer could have circulating tumor cells that are HER2^+^, or that trastuzumab may have off-target effects on other RTKs or other pathways connecting to PI3K/AKT. Although these mechanisms remain to be explored, the findings of abnormally high HER2 signaling activities in a subset of HER2^−^ tumors offer an alternative explanation for why some patients with HER2^−^ breast cancer derive benefit from HER2 signaling inhibitors. The data presented here suggests that an alternative test for HER2 status based on HER2 functional signaling activity (such as the CELx HSP test) could be useful for accurately determining patient eligibility for HER2 signaling inhibitors to maximize the therapeutic benefit.

Studies have suggested that upregulation of HER2 signaling in HER2- patients is caused by activating mutations in the extracellular or kinase domains of HER2 receptor [28]. In one of the largest clinical studies of HER2 mutations, Ross et al. report that in 5,605 cases 10.6% had ERBB2 amplification, 2.4% had ERBB2 mutation where 0.7% had co-occurring ERBB2 amplification and mutation with only about 11% of all mutations found affecting the extracellular (ligand binding and dimerization) domain of the receptor [29]. This very low percentage (2.4% × 11%) is significantly less than the 20% level inferred from the 34 patient results reported in the present manuscript (20.5%; 95% CI = 10%–37%). While HER2 mutation may explain some HER2- patients' dysfunctional HER2 signaling, it could likely explain, at most, one of our results. Additionally, only costly DNA sequencing can uncover the mutants and the functional implication of each and combinations thereof are still under investigation [30]. Thus, we believe our approach provides a more complete and thus applicable method to measuring HER2 signaling dysfunction in HER2- samples.

Previous studies using molecular and biochemical methods for refined assessment of HER2 levels and HER2 phosphorylation status have suggested that HER2 phosphorylation above a threshold occurs in a subgroup of HER2^−^ breast tumors [31–35]. In one of these recent studies using retrospective protein microarray analysis, the phosphorylation of HER2 and signaling proteins downstream of HER2 were measured. The report concluded that a portion (8–16% of 140 samples in two groups) of the tested HER2^−^ samples had phospho-HER2 levels correlated with phospho-HER2 levels of HER2^+^ samples. However, the status of the PI3K/AKT and MAPK pathways, the two main effector pathways downstream of HER2, were not reported, functional activation of meaningful disease markers were not apparent, and discrepancies existed between methods used to prepare the samples for analysis [35]. Santarpia et al. [36] recently review biomarker studies in breast cancer and conclude: “It is likely that it is the combined effect of all genomic variations that drives the clinical behavior of a given cancer. Furthermore, entirely new classes of oncogenic events are being discovered in the noncoding areas of the genome and in noncoding RNA species driven by errors in RNA editing. In light of this complexity, it is not unexpected that, with the exception of HER2 amplification, no robust molecular predictors of benefit from targeted therapies have been identified.”

Typically, the classical endpoint assays used in these studies provide incomplete, static information on HER2 activation (i.e., phosphorylation at a limited number of sites on a limited number of targets, requiring *a priori* knowledge of all the potential dysfunctional processes) that may be somewhat correlative but may not be causally related to disease activity, drug function, and the status of the HER2 signaling network. Allosterism, differential phosphorylation/dephosphorylation, phosphorylation density, scaffolding assembly, signaling crosstalk, bistability or ultrasensitivity [37], and a myriad of mechanisms of drug resistance may also contribute to the quantitative and qualitative activity of the HER2 signaling pathway and all contribute to the difficulty in using a static gene or protein measurement to comprehensively quantify signaling pathway regulation that relates to drug response and therapeutic outcome prediction [38–40]. In contrast, using the CELx HSP test, data strongly suggests in this set of HER2^−^ tumors that HER2–HER3 hetero-dimers are the main driver of abnormally high ligand-dependent HER2 signaling. Furthermore, in the present study, the CELx HSP test provides real-time functional monitoring of patient cell response to agonist (ligand) and antagonist (drug) in order to provide a more complete method of diagnosing HER2-driven disease than the HER2 protein or genetic assays currently used in the clinic. This is the first study using diseased and healthy cells from patients *ex vivo* in a clinical test format for direct assessment of the functionality of the disease-related signaling pathways and determination whether a drug therapy has the intended effect on those cells.

The present results would favor a hypothesis that if the mechanism of a disease, target activity, and drug response are all present in a patient's cells *ex vivo*, the chance that the patient will respond to a targeted therapy will be increased. The scenario *in vivo* could be more complex when considering variables such as immune system complexity and heterogeneity of the tumor microenvironment.

The approach described in this work suggests a path to developing more precise, personal, cancer diagnoses and treatment. In clinical use, the test would be performed as a CLIA/CAP certified Laboratory Developed Test (LDT) or an FDA cleared or approved *in vitro* diagnostic. The test itself would be performed in a central laboratory that would process the specimens, perform the test, and provide the result to the ordering clinician. Prior to becoming available for clinical use, the CELx HSP test must first be analytically validated to confirm that the sensitivity and specificity of the test meets appropriate requirements, balancing the relative patient risk posed by a false positive versus a false negative result. Once analytically validated, a prospective clinical trial designed to evaluate whether HER2-negative patients reported to have abnormal HER2-driven signaling using the CELx HSP test respond to treatment with anti-HER2 therapies would be performed.

The authors developed and validated methods to collect tumor tissue at a clinical site and have it delivered overnight (< 24 hours) in fresh condition to a central test laboratory. These methods, used in the present study, involve placing the specimen immediately after removal from the patient in a glucose-based solution designed to maintain the tissue in fresh condition and shipping it overnight in a kit designed to maintain temperature below 8°C to a central test laboratory. For this and other related studies, clinical sites providing tissue specimens demonstrated 98% compliance with this collection protocol (*N* = 150). In the few cases where the collection protocol was not followed, proliferative cells were obtainable from 50% of the non-compliant collection, confirming the robust nature of these methods.

By analyzing the signaling activity in a patient's live tumor cells, signaling abnormalities driving a cancer can be revealed that a genomic or proteomic test has not detected. The present data show no correlation between protein expression level and signaling activity in ER^+^/HER2^−^ primary cells. These results suggest a new subtype of breast cancer – HER2^−^ (receptor expression normal)/HER2^Signal+^ (signaling abnormal). If confirmed in future work with accredited validation studies, mouse xenografts, larger prevalence studies, and interventional studies of HER2^−^ patients treated with HER2-targeted drugs, this discovery would have significant clinical implications. Patients diagnosed with this new subtype of cancer would potentially benefit from anti-HER2 therapy. The CELx HSP test has the high potential to be developed into a clinical diagnostic test or confirmatory test for better informing physicians about HER2-driven diseases such as breast cancer or HER2-driven cancer in other tissues [41, 42]. Additionally, this novel method of identification of new cancer subtypes can drive development of new drugs or repurposing of existing drugs. This tumor assessment technology may change the way personalized medicine is practiced in the future.

## MATERIALS AND METHODS

### Specimen

Healthy and tumor specimens were obtained from excess resected human breast tissue from women over 18 years of age undergoing standard-of care therapeutic surgery and histological diagnosis. Tissues and supporting information were de-identified prior to delivery to Celcuity. Liberty/Chesapeake IRB (Columbia, MD) determined that this research did not involve human subjects as defined under 45 CFR 46.102(f) and issued a written IRB exemption.

Fresh specimens were obtained from the University of Minnesota, Clinical and Translational Science Institute Biological Materials Procurement Network, (Minneapolis, MN) and Capitol Biosciences (Rockville, MD). All specimens were immersed in organ transplant solution within one hour of excision. The specimens from the University of Minnesota were shipped overnight in containers shown to maintain 0°C–8°C for 36 hours. The specimens from Capitol Biosciences were frozen as described further below and then transported on dry ice.

The 34 tumor tissue specimens included in this study were from pre- and postmenopausal patients previously diagnosed with HER2^−^ breast cancer at different clinical stages of advancement and with diverse histologies and lymph node status (Table 3). *A priori* exclusion criteria for healthy tissue specimens were as follows: pathogenic viruses – HPV, HIV, HBV, HCV, HAV; benign cysts, phylloid/phyllodes tumor, or other non-cancerous abnormality; current or previous diagnosis or treatment for any cancer, diabetes, or thyroid disease. In addition, specimens from patients with known BRCA1 or BRCA2 gene mutations were excluded to prevent inadvertent inclusion of patients who may have undiagnosed cancer. A de-identified copy of each patient's pathology report was provided to the authors. The different sections of the tumors were evaluated by a pathologist using standard clinical IHC protocol and those samples included in this study were HER2 0 or 1 (i.e. samples with HER2 2+ or HER2 3+ IHC scores were excluded) Tumor tissue specimen were collected from two providers as available between May 2013 and June 2015. The 34 tumor and 16 healthy samples in this study were tested over approximately a four-week period.

**Table 3:** Summary of patient characteristics

Characteristic	No.	Percentage (%)
No. of breast cancer patients	34	
Age, years		
Mean	57.5	
36–60 years old	18	53
61–79 years old	16	47
Clinical Stage		
I	5	15
II	22	65
III	5	15
N/A*	2	6
Histology		
Invasive only	13	38
Invasive Ductal/DCIS mixed	11	32
Lobular/other	8	24
N/A*	2	6
Lymph Status		
Metastatic	12	35
Not Metastatic	20	59
N/A*	2	6
Estrogen Receptor Status		
ER+	26	76
ER–	1	3
N/A*	7	21
HER2 IHC score/FACS		
0/1+	34	100
2+/3+	0	0

* Information not available due to nature of some de-identified surplus tissue used in this study.

### Cell culture

Methods for tissue extraction, primary cell culture, and short-term population doublings are essentially as described previously [43, 44]. Briefly, 20–70mg tissue was minced with scalpels to < 2-mm pieces and cryopreserved until testing (Unisol, Cell and Tissue Systems, Charleston, SC) [45] or used fresh. Tissue (20–40 mg) for CELx HSP testing was enzymatically disaggregated for minimal time to obtain cells and cell clusters in collagenase and hyaluronidase (Worthington Biochemical, Lakewood, NJ) at 37°C in 5% CO2. The disaggregated tissue was washed in culture media to remove disaggregation enzymes, plated on 6-well tissue culture plates in serum-free mammary epithelial cell media, and grown until approximately 2 × 10^5^ cells were available. Trypan blue staining was used before initial plating to determine the *a priori* viability of each specimen. After four days in culture, media was changed regularly.

The DAKO IHC test standard breast cancer cell lines SKBr3 and MDA-231 were maintained according to provider's instructions. SKBr3 was grown in McCoy's 5A supplemented with 10% FBS and 1% penicillin/streptomycin/amphotericin (PSA). MDA-231 was grown in Leibovitz's L-15 Medium with 10%FBS and 1%PSA. All cell lines used in this study were authenticated by ATCC in March 2016 by comparison with the ATCC short-tandem repeat (STR) databases.

### Flow ctometry

Antibodies used in this study are detailed in Table 4. Flow cytometry was performed on a BD FACSCalibur (BD Biosciences, San Jose, CA) using cells harvested at the time of CELx HSP test using methods previously described by others [46]. Data were analyzed with FlowJo 2 software (FlowJO, Ashland, OR). The flow cytometry studies conducted for this work produced results 100% concordant to the standard clinical IHC test evaluations for HER2, ER, and PR that were provided for each specimen by the clinic that provided the specimen. No samples with any HER2 overexpressing cells were included in this study.

**Table 4:** Antibodies used in this study

Description of Antibody	Vendor
mouse anti-human HER2-phycoerythrin (PE), clone 24D2	Biolegend, San Diego, CA
mouse anti-human HER1 conjugated with AlexaFluor 647, clone EGFR.1	BD Biosciences, San Jose, CA
mouse anti-human HER3 conjugated with PerCP-sFluor710, clone SGP1	eBioscience, San Diego, CA
mouse anti-human EPCAM conjugated with AlexaFluor 488, clone MH99	eBioscience, San Diego, CA
mouse anti-human Claudin4 conjugated to PE, clone 382321	R&D Systems Minneapolis, MN
rat anti-human CD49f conjugated to PerCP/eFluor710, clone eBioGoH3	eBioscience, San Diego, CA
mouse anti-human CD10 conjugated to Allophycocyanin (AP), cloneHL10a	BioLegend, San Diego, CA
rabbit polyclonal anti-human estrogen receptor alpha (ERα) conjugated to AlexaFluor488	Bioss, Woburn, MA
mouse anti-human progesterone receptor (PRG) conjugated to eFluor660, clone KMC912	eBioscience, San Diego, CA

*All epitopes were extracellular with the exceptions of ER and PR. All antibodies were purchased from companies as listed who provided empirical demonstration of each of the antibodies for our applications.

### CELx HER2 signaling profile (HSP) test

HER2 signaling network activity was measured using the xCELLigence RTCA (ACEA Biosciences, San Diego, CA) as described elsewhere [11]. Briefly, cells were seeded in triplicate wells of 96-well sensor plates (pre-coated with collagen and fibronectin) in serum-free minimal medium (assay medium) the day before growth factor treatment. Impedance values were recorded throughout the whole course of an experiment. Drugs and inhibitors were added into the sensor plates at least eight hours prior to the addition of growth factors. Growth factors were added 18–24 hours after cell seeding. All reagents were dispensed with a VIAFLO automatic liquid handler (Integra Biosciences, Hudson, NH).

An NRG1b titration series from 375 pM to 12 nM and an EGF titration series from 25 pM to 800 pM were performed during assay development. A single dose of 3 nM for NRG1b and a single dose of 0.3 nM for EGF were chosen for CELx HSP testing of all samples that represented a maximal signal plateau for samples with different levels of HER receptors.

### Data analysis and statistics

Data were exported from the RTCA for analysis by TraceDrawer (Ridgeview Instruments, Sweden) and Microsoft Excel. Impedance time course data was recorded for one of three categories of each cell sample tested: cells with addition of media only (C), cells with addition of growth factor stimulus only (CF), and cells with addition of an antagonist drug (HER2-specific dimerization blocker) followed by a growth factor stimulus (CDF). To permit inter-sample quantitative comparison, CI values were set to zero for each set of CI time course data at the time stimulus was added to a cell sample. By convention, the manufacturer converts impedance to cell index units (Ci = Ω/15). After the stimulus was added, data were assessed by one of the following algorithms. To determine the gross HER2 signaling activity resulting from growth factor stimulation, the formula CF–C was used. The signal accompanying the addition of each growth factor arises from hetero-dimerization that may occur if HER2 participates and any homo-dimerization of the receptor. To determine the net HER2 participation in HER signaling initiated by a growth factor, the algorithm [(CF-C)-(CDF-C)] was used, where the values associated with the EGF and NRG1b stimulus and inhibition were combined to arrive at a total amount of HER2 signaling for a particular cell sample.

To classify a HER2^−^ sample as having abnormal signaling, a test cut-off value of 250 was established that was 90% of the upper range of the healthy responses and coincidental with the median test value for the sample set of HER2^+^ cell lines previously tested [11]. To confirm with 95% confidence that at least 5% of HER2^−^ patients have abnormal pathway signaling, assuming that 15% of the population would be abnormal, a sample size of 33 was required.

To confirm cells from normal patients and those from the HER2^+^ cell lines generate different HER2 signaling levels using the CELx HSP test, it was first assumed that normal breast cells have a median CELx HSP test value of 100 with a standard deviation of 100. To detect a 150 absolute difference (assuming 250 for HER2^+^ nd 100 for normal) in test value between the two types of cells, with a two-sided α at 0.05 and 90% power, the required number of patients in the normal population and HER2^+^ population arms is 10 each. Since only nine HER2^+^ positive cell lines were available, the sample size of the normal specimens was increased to 16 to offset the effect of the smaller HER2^+^ sample size.

Pearson correlation analysis was performed with GraphPad Prism 6 to evaluate the relationships among the variables of interest. All dose-response curves were obtained using nonlinear regression curve fitting with GraphPad Prism 6. Box-and-whisker plots were constructed to analyze the different groups' CELx HSP test results. *P* < 0.05 was considered statistically significant.
